# LED Technology Applied to Plant Development for Promoting the Accumulation of Bioactive Compounds: A Review

**DOI:** 10.3390/plants12051075

**Published:** 2023-02-28

**Authors:** Oana Livadariu, Carmen Maximilian, Behnaz Rahmanifar, Calina Petruta Cornea

**Affiliations:** 1 Faculty of Biotechnology, University of Agricultural Sciences and Veterinary Medicine of Bucharest, 59 Bd. Marasti, 011464 Bucharest, Romania; 2 Institute of Biology Bucharest of Romanian Academy, 296 Spl. Independentei, 060031 Bucharest, Romania

**Keywords:** LED, flavonoids, phenols, carotenoids, terpenes, glucosinolates, food preservation

## Abstract

Light is an important environmental factor for plants. The quality of light and the wavelength stimulate enzyme activation, regulate enzyme synthesis pathways and promote bioactive compound accumulation. In this respect, the utilization of LED light under controlled conditions in agriculture and horticulture could be the most suitable choice for increasing the nutritional values of various crops. In recent decades, LED lighting has been increasingly used in horticulture and agriculture for commercial-scale breeding of many species of economic interest. Most studies on the influence of LED lighting on the accumulation of bioactive compounds in any type of plants (horticultural, agricultural species or sprouts) and also biomass production, were carried out in growth chambers under controlled conditions, without natural light. Illumination with LED could be a solution for obtaining an important crop with maximum efficiency, with a high nutritional value and minimum effort. To demonstrate the importance of LED lighting in agriculture and horticulture, we carried out a review based on a large number of results cited in the literature. The results were collected from 95 articles and were obtained using the keyword LED combined with plant growth; flavonoids; phenols; carotenoids; terpenes; glucosinolates; food preservation. We found the subject regarding the LED effect on plant growth and development in 11 of the articles analyzed. The treatment of LED on phenol content was registered in 19 articles, while information regarding flavonoid concentrations was revealed by 11 articles. Two articles we reviewed debate the accumulation of glucosinolates and four articles analyzed the synthesis of terpenes under LED illumination and 14 papers analyzed the variation in content of carotenoids. The effect of LED on food preservation was reported in 18 of the works analyzed. Some of the 95 papers contained references which included more keywords.

## 1. Introduction

Light is one of the most important physical factors involved in the growth and development of plants, especially in the photosynthesis process. The quality and wavelength of light determine the synthesis of primary (proteins, carbohydrates, vitamins, etc.) and secondary (polyphenols, flavonoids, anthocyanins, lignin) metabolic compounds. These bioactive compounds confer certain properties to the cultivated horticultural or agricultural species such as taste, appearance, smell and quality. 

However, not all light spectrum is beneficial; plants generally absorb radiation in the visible spectrum. Apart from photosynthesis, light influences flowering time and morphogenesis. Light is absorbed by photoreceptors, namely, the phytochromes (absorb far-red/red light) and cryptochromes (absorb far-red and blue light and ultraviolet A light) are responsible for plant morphological and developmental changes [1,2].

In view of these aspects, attempts have been made over the years to illuminate with a certain wavelength to improve the quality of the crops obtained in greenhouses and in the warehouses where they are stored. That is why the application of LED artificial light was the most important step in the development of technologies that allow illumination with a certain wavelength, which has brought benefits in the control of the growth and development processes of plants and crops. LED irradiation has been found to be effective in stimulating plant metabolite production after harvest and during development [3]. Many previous studies have demonstrated the effective use of LED lighting in in vitro growth and organogenesis of plants. The aim of the paper is to release an extended overview of the most important and recent data regarding the effect of LED treatments in the field of horticultural and agricultural research for obtaining healthy plant biomass with a better nutritive quality, especially in the accumulation of bioactive compounds.

## 2. The History of LED Discovery

The history of the invention of the LED, light emitting diode, dates back to some of the earliest days of wireless technology—a time when little was understood about semiconductors themselves and even less about the possibility of using them to generate light. LEDs have been commercially available since the 1960s, but the LED history extends many years before this—the LED invention has its roots in much earlier developments. [4]

Captain H. J. Round, a British radio engineer was the pioneer of the history of the LED and the first person who noticed the LED effect. The first recorded effects of the light emitting diode were observed back at the beginning of the twentieth century. H. J. Round, who working for Marconi, undertook some experiments using crystal detectors. At the time, radio detectors were one of the major limiting factors within the early wireless radio sets. 

However, the first LED was created in 1927 by Russian inventor Oleg Lossev who published the theories of his discovery. He had published a total of four patents between 1927 and 1942; his first paper was entitled: “Luminous carborundum detector and detection crystals”, which was published in a Russian journal and then a series of experimental papers; although what was considered as junction electroluminescence in a semiconductor was first published in 1907 by H. J. Round. Unfortunately, all this work published by Lossev was lost and destroyed in Leningrad. He lived in Leningrad and was killed during the siege of Leningrad during the Second World War.

In 1951, a better understanding of LED operation was provided by K. Lehovec and his colleagues, following the development of p-n junction theory. Following papers published by Haynes and his colleagues in 1952 and 1956, the light-emitting potential of LEDs began to be considered and applied to light bulbs. However, up to that point, the light was a very red light, so a material had yet to be found to create a white or warm white/yellow light [5]. The development of a suitable LED light for traditional light bulb replacement took a long time and even when a suitable white light or color temperature was found the light intensity or brightness was too low. Subsequently, the application of LEDs remained in solid state or electronic applications.

In 1962, the first practical visible spectrum LED light was developed by Nick Holonyak Jr. from General Electric. Then, in 1972 Holonyak’s graduate student, M. George Craford invented the first yellow LED and 10x brighter red and red-orange LEDs. When green LEDs then came along, the applications increased with the use of three basic colors.

The breakthrough occurred in 1994 when Shuji Nakamura and colleagues from the Japanese company Nichia developed a blue high brightness LED light. This was significant for a number of reasons but also because when blue, green and red LED light are added together the light appears white to the human eye. Nakamura’s invention formed the basis for future white LED light production and he was awarded the Millennium Technology Prize in 2006. However, it was not until approximately 2008 when LED light output was enough to produce a light of sufficient intensity to use in residential and commercial lighting.

The first red light-emitting diode (LED) was developed in 1962. The first use of LEDs in studies of plant growth and development was made two decades later, in 1988. To this goal, NASA developed a project to install LED-illuminated plant growth and development systems in space. It was not until 2000 that Japan became the first country to use LEDs commercially for plant production. Subsequently, the use of LEDs in this field, with their multiple applications in horticulture, has gained widespread use.

In the present, LEDs have many applications in agriculture, horticulture and in crop preservation. Moreover, most studies on the influence of LED lighting on the accumulation of bioactive compounds in plants, and also biomass production were carried out in growth chambers under controlled conditions, without natural light. 

The advantages of using LED technology are: ideal light sources in “plant factories”, presenting high lighting efficiency by positioning close to the plants but giving off little heat (low heat generation), ensuring lower energy costs for lighting; providing uniformity of lighting—the LED is a microchip that can be easily positioned according to requirements, having low power consumption, long lifetime, safe handling (no risk of breakage when in contact with water droplets and easy dust cleaning), small size, durability, emitting cold light (climate control for greenhouse grown plants) and the possibility of wavelength selection to achieve the desired process modulation; color can be selected without adding filters and maintaining illumination intensity; customized wavelengths compared with other artificial light sources.

## 3. The LED Effect on Plant Growth and Development 

Plant growth and development are affected by certain wavelengths of light, especially red and blue light which are considered to be the most important [6,7,8].

The red light promotes the growth of wheat seedlings [9], mainly manifested by increased plant height, leaf area, shoot fresh weight and dry weight per plant under white + red LED illumination. They determined the effect of white (W), white + red (WR), and white + blue (WB) light-emitting diode (LED) treatments on the growth, nutritional characteristics and antioxidant properties of wheat seedlings. Similar results were reported [10] in water spinach which ascertained that the red LED treatment caused a massive increase in fresh stem weight and plant height. In contrast, blue LED light in combination with white LED light had a negative effect on wheat growth, as reported by other studies [11]. This could be due to the fact that the high proportion of blue light from WB LEDs inhibited stem elongation and leaf surface expansion [12]. However, the effect of red and blue light on the growth of wheat seedlings diminished over the time, which indicated that this effect might be related to the growth stage of the wheat or with an acclimatization response to new light conditions.

**Figure 1:**
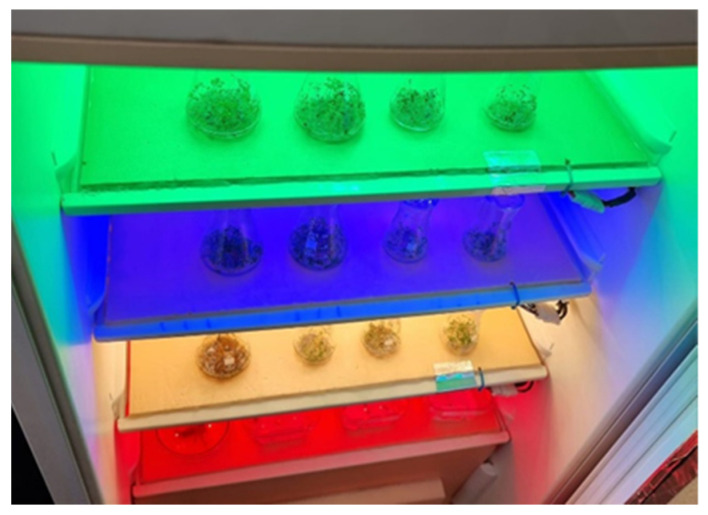
Aspects of LED experiment laboratory (Photo original Livadariu O.).

Other researchers [13] determined the influence of treatments with red, white or blue LEDs on the rate and the fresh weight of wheat sprouts. The sprouts were obtained from two commercial varieties (S1 and S2). They observed that red LED induced the highest rate of S1 sprouts while white LED produced the lowest rate of S1 sprouts. The white LED produced the highest influence on S2 sprout rate, while the red LED inhibited the rate of S2 sprouts. The fresh weight for S1 was increased by treatment with red LED whereas white LED decreased it. The highest weight value of sprouts was obtained by illuminating with blue LED and the lowest value was induced by red LED in the case of the S2 variety. Sprouts treated with three types of LED spectrum variants red (R), blue (B) and green (G) showed that blue (B) LEDs determined a high level of growth rate and fresh weight of hemp (*Cannabis sativa* L.) sprouts [14]. Another paper [15] carried out a study regarding the effect of treatment with white, red, blue LEDs on the growth rate and the fresh weight of two varieties of mung bean (M1 and M2) (*Vigna radiata* L.) sprouts and the results demonstrated that the treatment with blue LED induced a superior number of sprouts in comparison with red and green variants of LED (Figure 1). The highest value of fresh weight of mung bean sprouts was obtained for those illuminated with red LEDs obtained from M1 seeds. Similar research reported the influence of illumination with white (W), red (R), blue (B) and green (G) LEDs on the rate and the fresh weight of garlic (*Allium sativum* L.) sprouts [16]. Experimental results indicated that blue (B) LED determined an increase in the growth rate of garlic sprouts, while white (W) LED an improvement in the fresh weight of garlic sprouts.

*Schefflera arboricola, Dracaena godseffiana, Philodendron selloum, Syngonium podophyllum* and *Scindapsus aureus* are five indoor ornamental species which are effected by irradiation with LED [17]. An adequate light is an important factor for growth and development. An LED illumination of 1500–1900 lux produced a significant increase in length in *Schefflera arboricola*; *Dracaena godseffiana* showed a high value of plant height under 1500–1900 lux, plant height, spread and leaf thickness were achieved in *Syngonium podophyllum* under 1100–1500 lux and 1500–1900 lux, while *Scindapsus aureus* showed maximum plant height, spread, leaf area and leaf number when treated with 1100–1500 lux and *Philodendron selloum* presented maximum plant spread under 700–1100 lux.

The four LED light treatments applied to two *Ocimum basilicum* cultivars (Lettuce Leaf and Red Rubin-mountain Athos hybrid): AP673L (high red and high red:far-red), G2 (high red and low red:far-red), AP67 (moderate blue and red and low red:far-red), and NS1 (high blue and green, high red:far-red and 1% ultraviolet) with different colors mixing UV, blue, green, red and far-red and fluorescent tubes (FL, high blue, green and red:far-red) as control, noticed that the root lengths of Lettuce Leaf were higher under AP673L compared to NS1 and total biomass was significantly greater under NS1, AP67 and G2 compared to the control, for both cultivars [18]. Seedlings of Lettuce Leaf cultivated under the effect of the control and AP673L, and seedlings of Red Rubin hybrid grown under AP673L (mainly) quickly developed new root system.

Summarizing the results of these investigated articles, it is possible to make an analysis of the growth and development parameters determined and the effect of LED lighting, depending on the spectrum applied. Thus, the plant growth parameters analyzed were the following: leaf area: five articles; sprout rate: five articles; sprout fresh weight: five articles; shoot dry/fresh weight: three articles; shoot fresh/dry height: one article; plant height: two articles; leaf numbers: two articles; leaf thickness: two articles; leaf length: one article; leaf weight: one article; root length: one article; root growth capacity: one article; root density: one article; root weight: two articles; plant spread: one article; chlorosis: one article; flower buds: one article. For these parameters, treatments with white LED were applied in four articles, blue LED in six articles, green LED in two articles, red LED in six articles, and a combination of white + red LED in one article, white + blue LED in one article or high red and high red:far-red, high red and low red:far-red LED, high blue and green LED, high red:far-red and 1% ultraviolet, moderate blue and red and low red:far-red LED in one article. A single article debated the effect of LED light intensity on plant growth, without mentioning the wave length, only the light intensity.

All these results regarding the effect of LED irradiation are represented in Table 1.

## 4. The Effect of LED Application on Synthesis of Secondary Metabolites and Bioactive Compounds

### 4.1. Phenols

Numerous studies on the effect of LED lighting on the content of some secondary metabolites have been conducted to explain their mode of action in the metabolic pathway for the synthesis of these compounds. Specialized metabolites play a fundamental role in the aromatic function of several herbs, promoting both the characteristic odors and flavors [19].

Thus, the content in bioactive compounds was enhanced by treatments with a combination of red, green and blue LEDs in equal proportions. This treatment was performed on two varieties of *Ocimum basilicum*: Dark opal and Caesar [20] leading to an increase in anthocyanin, phenol and carotenoid content. In addition, the increase in phenol and carotenoid synthesis was enhanced by illumination with a combination of LEDs in which the red component was four-fold higher. A red basilicum treated with a 2.3:1 red:blue combination supplemented with white LED resulted in a significant increase in anthocyanin content. Similarly, illumination with LEDs in the same ratio and combination resulted in an increase in phenol concentration in green basil [21]. The same experiments performed on a green cultivar illuminated with blue LED supplemented with white LED did not stimulate phenol synthesis. In fact, no effect was observed after 16 h per day exposure of blue light for different periods (0 to 48 days) on the phenol content of basil. The authors also reported that light intensity (300 μmol m^−2^ s^−1^) would be an important factor in inducing antioxidant synthesis [22]. 

Other experiments performed on basil stated that the highest phenol concentration was synthesized in response to red and blue light in the ratio R:B = 2:1 or 3:1 [23], which supports the hypothesis that an increase in the red component in the mixture may enhance antioxidant production [24]. In contrast, light intensity (e.g., in the range from 100 to 300 μmol m^−2^ s^−1^) did not increase phenolics in basil leaves under red and blue irradiation (R:B = 3:1) [25].

The red LED light treatment applied 16 h/day caused an increase in the accumulation of phenols compared to monochromatic blue, green and white light in *Allium sativum* [16]. In the case of *Coriandrum sativum* species, monochromatic blue LED illumination induced the most significant increase in phenol content compared to monochromatic red and green LED, as well as red and blue, and red, blue and far-red combinations [26]. An increase in polyphenol and flavonoid content was observed [16] under the red LED treatment, while the blue LED treatment inhibited their synthesis. Antioxidant activity was enhanced under the action of blue LEDs and inhibited by white LEDs; the experiments used white, blue and red monochromatic LED. In both *Allium sativum* and *Coriandrum sativum*, monochromatic green light appeared to have a limited effect on phenol accumulation in plants. 

Green monochromatic LEDs did not induce significant effects in the accumulation of secondary metabolites, but research on hemp sprouts [14] showed that antioxidant activity was high with green monochromatic LED light compared with red or blue while illumination with blue (B) LEDs determined the amplifying of the metabolic pathways for the biosynthesis of flavonoids and polyphenols. However, there is not enough literature investigating the effect of this type of LED; therefore, further studies are required [27]. The simultaneous application of drought stress and a red and blue light (R:B = 2.3:1) to melissa (*Melissa officinalis*) resulted in an increased content of phenolic compounds [28]. It is possible that drought stress and ROS formation [29] could be balanced by an increase in antioxidant production, stimulated in particular by R:B treatment [28]. 

The highest concentration of polyphenols in wheat sprouts was determined by irradiation with sunlight, while the enhanced antioxidant activity was induced by illumination with blue (B) LED [13]. 

Considering the 19 works on the effect of LEDs on phenol content, we can say that both monochromatic LEDs (red or blue LED) and combinations of different LED spectra induce positive effects. The beneficial effect of red LED illumination on phenol compounds was reported in six articles. We also found the best results on the accumulation of phenols by irradiation with blue LED in six papers. Some papers (six) offered data regarding the use of the combination of different spectra (red, blue or green) which induced high phenol concentrations. A single paper presented data on the accumulation of phenols under the action of white LED.

### 4.2. Flavonoids 

Flavonoids are widespread in plants and involved in multiple cellular mechanisms, including protection against pathogens and ultraviolet (UV) radiation, plant coloration and male fertility [30,31,32], protection of leaf cells against photooxidative damage [33], response to oxidative stress [34,35]. Numerous previous studies have reported the key role of LED in flavonoid biosynthesis in plants. 

Accumulation of flavonoid compounds in leafy greens illuminated with supplemented white, blue (440 nm) and red (660 nm) LEDs in green- and purple-leafed sweet basil (*Ocimum basilicum* L.), lamb’s lettuce (*Valerianella locusta* (L.) Laterr.) and garden rocket (*Eruca sativa* L.) was reported recently [36]. In this paper, a non-destructive method was used using an optical sensor for evaluation of flavonol, anthocyanin and chlorophyll indexes in plants and reported that blue LED induced a significantly increased flavonoid index in both green- and purple-leafed basil, lamb’s lettuce and garden rocket plants.

The data reported in the studies on *Triticum aestivum* sprouts regarding the treatments with sunlight, red, white and blue LEDs revealed the fact that the influence exercised by LEDs emitting red (R) light improved the flavonoid concentration [13].

Blue LED irradiation produced the accumulation of high amounts of anthocyanins in grapes [37] and the upregulation of VIMYBA1-2 genes, VIMYBA2 and VvUFGT, involved in anthocyanin biosynthesis. Similarly, other studies observed increased anthocyanin accumulation in buckwheat seedlings treated with blue LED [38]. Experiments on buckwheat sprouts illuminated with blue LED demonstrated a wide variation of FtPAL, FtANS genes and FtDFR expression involved in the biosynthesis pathway of flavonoids. Further research on *Cyclocarya paliurus* (wheel wingnut) illuminated with blue LEDs reported a positive correlation between anthocyanin accumulation and the expression of genes involved in flavonoid synthesis, including the 4-coumaryl CoA ligase (4CL) and phenylalanine ammonia synthase [39,40] in research conducted in *Agastache rugose* (Korean mint) illuminated with white LEDs, revealed an important accumulation of two phenylpropanoids compounds: rosmarinic acid (antibacterial, antiviral, antioxidant, and anti-inflammatory properties) and tilianin (antihypertensive, anti-inflammatory, vaso-relaxant and antiatherogenic effects). reported that white LED (380 nm) was the optimal wavelength for epicatechin biosynthesis in wheat sprouts, while blue LED (470 nm) enhanced the accumulation of gallic acid and quercetin, but decreased the levels of p-coumaric acid and epicatechin; red light (660 nm) increased the accumulation of ferulic acid at 8 days and p-coumaric acid at 12 days [41]. Research on *Anoectochilus roxburghii* (Jewel orchid) treated with a combination of blue and red LEDs in a 1:4 ratio showed an increased expression of the genes involved in flavonoid synthesis (phenylalanine ammonia lyase (PAL), chalcone synthase (CHS), flavonol synthase, which led to increased flavonoid accumulation [42].

Thus, we can conclude that high flavonoid concentration was reported in 11 articles as follows: illumination with red LED—two papers, blue LED—five articles, three articles debated the effect of the combination of LEDs (red and blue) and one article described the positive influence of white LED.

### 4.3. Glucosinolates

Along with polyphenols and flavonoids, another category of secondary metabolites is synthesized in significant quantities under the action of LED lighting. This is the case of glucosinolates, S-containing glucosidic compounds, which constitute a natural class of organic compounds and are derived from glucose and an amino acid present in *Brassicaceae*, *Euphorbiaceae*, etc. The interest in glucosinolates has increased due to the biocidal [43] and cancer chemopreventive activity [44] of their hydrolysis products (isothiocyanates, nitriles, thiocyanates, epithionitriles, and oxazolidines).

The investigations regarding the effects of different LED light wavelengths (blue + red (470 and 660 nm), blue (470 nm), red (660 nm), or white (380 nm)) on the growth and production of glucosinolates and phenolic compounds in *B. napus* sprouts was accomplished [45]. Thus, the total glucosinolate content in *Brassica napus* sprouts was not significantly different in plants irradiated with white, blue and red LED. The highest phenol content was recorded in blue LED irradiated sprouts, 1.33 times higher than the lowest level in red light-irradiated plants. These results demonstrate that blue LED light is effective in increasing the content of glucosinolates and phenolics in *Brassica napus* sprouts [45]. In addition, blue LED treatment has been shown to induced an important biosynthesis of carotenoid in citrus juice sacs in vitro [46] and in Chinese skullcap callus [47], as well as increased concentration of glucosinolates in broccoli sprouts compared to blue + red LED [48]. It has been reported that red LEDs promote higher levels of phenolic compounds in *Myrtus communis* L. in vitro [49] and carotenoid synthesis in citrus [50], while white LEDs produce the enhancement of carotenoid production in buckwheat sprouts [51]. In addition, it has been shown that phenol production does not differ significantly in common buckwheat (cv. Kitawase) and Tartary buckwheat (cv. Hokkai T8) sprouts treated with blue LED, red LED and blue + red LED [52]. Based on these reports, it appears that the influence of different LEDs on the production of natural products might be dependent plant species, cells, tissues and organs. 

These studies evaluated the influence of different LED spectra on glucosinolate compounds. The numbers of these works are correlated with each wave light and the effect of the LED light. The increase in glucosinolate content was registered in two papers under illumination with blue LED or a mixture of blue and red LED after analyses of the articles concerning this type of secondary metabolite under LED irradiation.

### 4.4. Carotenoids

Carotenoids (carotene and lutein) are present in most plants and green algae and are involved in light capture and the transfer of light energy to the center of photosystems [53,54]. Carotenoids also neutralize the effect of ROS (reactive oxygen species) formed in the case of excessive light flux, protecting the photosynthetic apparatus [55]. Moreover, the consumption of carotenoids has been associated with several health benefits in humans, including heart disease and cancer prevention [56,57,58,59]. 

Many studies have reported the effects of both spectral quality and light intensity on carotenoid biosynthesis in plants. Red LED irradiation increased carotene production in pea plants [60]. Other studies reported that red LED led to increased cryptoxanthin content in citrus [61] and lycopene content in tomatoes [62]. LED-irradiation duration affected carotenoid biosynthesis in growing plants. Irradiation with blue LEDs over a short period of time significantly increased the amount of carotene and violaxanthin in broccoli sprouts [48] compared to illumination with a blue and red LED combination. Other studies reported that the carotene and lutein levels in buckwheat sprouts were decreased by treatment with blue LED, compared with white LED irradiation [51]. The variation in content of carotenoids in LED-irradiated plants could be attributed to the differential expression of genes associated with carotenoid synthesis. For example, buckwheat sprouts grown under irradiation with different LEDs showed the overexpression of FtPSY, FtLCYB, FtCHXB, FtCHXE, FtLCYe and FtZEP, which are associated with carotenoid biosynthesis, treated with white LED [47].

The research carried out on a variety of *Lemon Basil* (*Ocimum* × *africanum*) showed that red and blue LED treatment induced carotenoid accumulation compared to monochromatic red LED [63]. The effect of LED light treatments on secondary metabolites has also been evaluated in other aromatic plants, with beneficial responses For example, in *Allium fistulosum*, the white LED treatment proved to be the most effective for carotenoid accumulation compared to blue, green, yellow or red LED treatment [64].

The study of articles on the effect of LED on carotenoid accumulation led to the following conclusions. The amplification of carotenoid compounds was observed after treatment with a combination of LED lights (red and blue, or red, blue, green) in two papers analyzed, while the blue LED irradiation induced a high carotenoid concentration in four articles. The red LED increased the carotenoids content in five articles viewed and three papers reported the beneficial effect of white LED.

### 4.5. Terpenes

The terpenes are some of the most widespread natural products, having many different functions in animals and plants, and by adding them to food, confer a specific flavor. For example, the flavors of lemon, cinnamon and many other spices are due to terpenes. Common terpenes are limonene and citral (present in lemons), camphor and pinene (pine), eugenol (garlic), anethole (sweet cumin, aniseed), thymol (thyme, oregano), geraniol (roses) and menthol. The highest amount of thymol (phenolic monoterpene) present in thyme and having antiseptic properties was extracted from *Thymus migricus* illuminated with blue LED [65]. Red LED produced a significant increase in lutein content in *Brassica oleraceae* [66] and phenylacetaldehyde in *Brassica oleracea* leaves [66] as well as in a *Petunia* hybrid [67]). Other researchers [68] found that red LED in combination with blue LED induced a four-fold increase in essential oil production in *Mentha longifolia*. The combination of blue, white and green LED induced increased antioxidant activity in rice leaves [69]. The antioxidant activity was higher with blue LED illumination compared to red LED and fluorescent light [70].

However, not only monochromatic LED illumination determines a high content in secondary metabolites (terpenes or terpenoids); also a combination of LEDs does the same. Research on *Anethum graveolens* (dill) has provided new information on the effects of LEDs on terpenoid accumulation. Thus, LED illumination with 70% red and 10% blue components induced an increase in terpenoid concentration [71]. In reference [65], they report that terpenoid content increases under the action of monochromatic red light in two *Thymus* species, namely *T. carmanicus* and *T. migricus*. Only in *T. migricus*, a combination of red and blue light (R:B = 2.3:1) increased terpenoid concentration, suggesting that metabolite biosynthesis may be associated with increased stress from red light exposure. A combination of red and blue light (R:B = 1:1.4, with blue at 435 nm) induced increased terpenoid content in basil [72], while monochromatic red and green LED treatments were more effective in increasing terpenoids compared to monochromatic blue, in *Mentha spicata* species [73]). 

In conclusion, from the analysis of these articles, we can state that the positive effect of a combination between blue and red LED light in a certain ratio was described in three papers reviewed and one paper debated the role of red and green monochromatic LED in inducing the accumulation of terpene compounds.

In Table 2 are summarized the most recent studies regarding the effect of LED irradiation.

## 5. The Effect of LED on Food Preservation, Postharvest Losses 

Besides their important role in plant growth and development and amplification of the synthesis of secondary metabolites and bioactive compounds, LEDs have applications as well as a non-chemical sanitation treatment that can be used as an alternative green technology for food preservation to minimize postharvest losses during storage and transport [75].

Thus, harvested red sweet peppers maintained the highest concentrations of β-carotene, chlorophyll and lycopene for 7 days [76] after exposure to red LED light. In addition, freshly harvested yellow and green sweet peppers exposed to red LED and freshly harvested red sweet peppers illuminated with blue LED accumulated phenolic compounds by potentiating the activity of the enzyme phenylalanine ammonia lyase (PAL), involved in the production of lignin precursors, flavanoids, and coumarins [76]. In avocado fruit, red LED enhanced epicatechin content by upregulation of PAL genes [77]. In tomatoes, red LED treatment improved lycopene and b-carotene content, total phenols and flavonoids during storage after harvest [78]. Moreover, blue LED treatment increased lycopene levels, chlorogenic acid, caffeic acid and rutin content after 7 days of storage [78]). In addition, red or blue LEDs improved the content of antioxidant compounds in fresh tomatoes.

The influence of red (630–640 nm) and blue (450 nm) LEDs on the changes in antioxidant constituents, activity, volatile compound, and overall acceptability of coriander leaves (*Coriandrum sativum*) during postharvest storage was monitored [79]. The results emphasized an insignificant increase in total phenolic compounds content by 9.34 and 6.39% on day 9 and a commercially allowable mass loss up to 9 days by exposure to blue LED and red LED. The leaves exposed to blue LED light for 2 h and stored for 3–9 days showed a reduction in color change and an increase in the antioxidant activities (DPPH, ABTS, and FRAP), quercetin content, and the concentration of typical coriander aroma, 2-tridecenal, 2-dodecenal and Z-9-19 hexadecenal (volatile compounds) on day 9. The authors noticed that the blue and red LED exposure had remarkably reduced the loss of total chlorophyll, while the b-carotene levels in the leaves exposed to the red LED light remained similar during the experiment.

Yellowing is the most evident symptom of senescence in green vegetables during postharvest storage [80] in the studies regarding the use of LED illumination for Brussels sprouts postharvest conservation. The yellowing caused by senescence induces chlorophyll degradation and leads to a loss of nutritional and commercial value or diminishes the yield. The authors investigated the effect of white-blue light-emitting diodes (WB LED) on the outer and inner leaves of Brussels sprouts during 10 d storage at 22 °C. They showed a lower respiration rate and retained the green color and visual quality, with more than 10 times the chlorophyll than the controls in outer leaves and 1.6 times in inner leaves at the end of the storage period. Moreover, the content of antioxidants was increased in outer leaves compared to inner leaves, while antioxidant capacity increased only in the outer leaves. Total flavonoid content was higher in outer than inner leaves, and about 20% higher in treated samples at day 10 of storage. The authors of [76,81,82] associated the delay in senescence with increased content of sugars in broccoli. The content in total soluble sugars decreased during storage, especially in outer leaves and the treatment with WB LED improving the carbohydrate levels. These results are similar to other papers carried out on lettuce varieties [83,84]. The conclusion was that WB LED lighting would be useful to maintain/improve the quality of Brussels sprouts for both storage and transport.

Maintenance the quality after harvest and protecting crops from pathogen attack remain challenges for horticulture and agriculture; LEDs have now gained attention as a useful tool for sustainable agricultural practices. For example, single-spectrum blue LEDs have limited postharvest decay caused by *Penicillium* species in citrus compared to those maintained under dark conditions [85,86]. In addition, combating fruit spoilage caused by pathogens has been observed through light-mediated stimulation of lipid signaling and subsequent accumulation of phospholipase A2, ethylene and octanal [87,88]. In addition, blue light can directly inhibit fungal sporulation and germination [89]. Therefore, blue light applied after harvesting could ensure crop protection by having a double effect: inhibition of fungal growth and stimulating host defense responses.

Red light compared to white fluorescent light inhibits lesion extension, inducing the expression of defense-related genes and also stimulates the synthesis of stilbene compounds [90]. Stilbenes acts as phytoalexins, playing a crucial role in plant defense against phytopathogens, as well as being involved in the adaptation of plants to abiotic environmental factors. In addition, increased synthesis of stilbenes, concomitant with the high expression of 16 genes responsible for the defense response, has been observed after exposure of plants to LEDs of different wavelengths [90,91].

Salicylic acid (SA) plays an important role in plant disease resistance. The mutants of red:far-red light photoreceptors are known to be compromised in SA signaling stimulation and resistance to *Pseudomonas syringae* [92]. Red LED light induces SA synthesis and the expression of SA-regulated PR-1 and WRKY genes in pathogen-inoculated cucumber plants [93]. Considering these two aspects, it can be stated that red light induces resistance in association with SA-mediated defense responses. Furthermore, the low red:far-red light ratio inhibits SA and jasmonic acid (JA)-mediated disease resistance in *Arabidopsis* by reducing the expression of SA- and JA-responsive genes. These results demonstrate that the jasmonic acid and salicylic acid-mediated effect can be correlated with LED ratio [94,95]. Different spectra of LEDs may activate different metabolic pathways that can be triggers in the reaction induced by defense hormones (e.g., SA and JA).

The results described in this last part of the review show a relationship between red, blue or a combination of white and blue LED-light exposure and postharvest storage time. The effect of red LED on improving crop preservation was emphasized in seven papers and six articles concerning blue LED light were included in the references. Four articles highlighted the positive role of illumination with a mixed white and blue LED light and three works using a blue and red combination for obtaining better preservation of crops. All these experimental data concerning the LED effect on food preservation during storage have been included in Table 3.

## 6. Conclusions

This review summarizes the potential of LED treatments to enhance specialized metabolite content and plant growth parameters. Some studies applied a combination of light spectra, especially red and blue, white and green light in different proportions, but the best performances were observed under monochromatic red or blue lights. where blue LED influenced chlorophyll formation and chloroplast development and also the accumulation of secondary metabolites, and red LED is involved in the mechanism of growth of biomass and plant production. Most experiments regarding the parameters of plant growth adopted an exposure time between 12 and 16 h/day (photoperiod). A large number of studies investigated plant growth combined with a variation of phenol, flavonoid, carotenoid, glucosinolate and terpene content. Among the specialized metabolites, many articles analyzed the phenol and flavonoid content, followed by carotenoids. Glucosinolates and terpenes were evaluated in only a few studies.

Because it is difficult to understand how plants respond to changes in LED quality, studies of the specific ratios in many different species and their responses are often contradictory. All these studies demonstrated the usefulness of using LED technology in the field of crop preservation and storage which is considered to be an effective strategy for accumulating health-promoting compounds to add health benefits for consumers.

The future for the application of LED in plant production lies in obtaining increased plant production in controlled environment conditions with reduced energy costs (using up to 85% less energy); obtaining sprouts (with an enhanced nutritive value and healthy qualities in a natural way) exclusively illuminated with LED light; producing crops to reach their full potential in a short time, in a continuous cycle. Considering the effects of climate change causing major reductions in agricultural production, the use of LED lighting technology in agriculture could be an extremely useful tool for obtaining constant, numerous annual harvests with almost no limitations, as the plants are constantly exposed to light, allowing faster growth. Innovations in the use of LED artificial lighting in plant breeding are fundamentally important and innovative. LEDs offer great cost savings and efficiency coupled with superb performance, making them one of the best choices for lighting plants. The new direction in the application of LED may be to obtain specific biological compounds with applications in medicine, cosmetics, etc., by the illumination of plants with specific wavelengths or the modification of a metabolic pathway for the production of a specific bioactive compounds.

## Figures and Tables

**Table 1:** LED treatment and the effect on plant morphological and developmental parameters analysis.

Species	Type of LED (Wave Length/Light Intensity)	Plant Morphological Parameters	References
*Triticum aestivum* L. wheat seedling	white + red (WR), white, white + blue (WB)	Leaf area, fresh and dry shoot weight, plant height	[9]
*Schefflera arboricola* *Dracaena godseffiana* *Philodendron salloum* *Syngonium podophyllum* *Scindapsis aureus*	1500–1900 lux 1500–1900 lux 700–1100 lux 1500–1900 lux	plant height, plant spread, leaf area, number of leaves/ plants, leaf length and leaf thickness root weight, root density, number of roots/plant root density	[17]
*Impatiens walleriana* (impatiens), *Salvia splendens* (salvia), *Petunia hybrida* (petunia), *Solanum lycopersicum* (tomato)	blue (400–500 nm) red (600–700 nm)	Leaf number, relative leaf area, fresh shoot weight, seedling height, dry shoot weight, leaf thickness. chlorosis score, flower bud number.	[11]
*Ocimum basilicum* cultivars (Lettuce Leaf, and Red Rubin-mountain Athos hybrid)	-high red and high red:far-red -high red and low red:far-red -high blue and green, high red:far-red and 1% ultraviolet -moderate blue and red and low red:far-red	root growth capacity shoot height, root length, leaf area, leaf dry weight, shoot dry weight, root dry weight	[18]
*Cannabis sativa* L. (hemp sprouts)	red, blue, white	sprout rate fresh weight	[14]
*Vigna radiata* L. (mung bean sprouts)	red, blue, green	sprout rate fresh weight	[15]
*Allium sativum* (garlic sprouts)	red, blue, white	sprout rate fresh weight	[16]
Water spinach (*Ipomoea aquatica*)	red, blue, green	sprout rate fresh weight	[10]
	red, blue, green	Stem weights, leaf fresh weights, shoot length, total leaf area/plant	
wheat (*Triticum aestivum* sprouts)	red, blue, white	sprout rate fresh weight	[13]

**Table 2:** The effect of LED on biosynthesis of secondary metabolites.

Species	Secondary Metabolites	LED Light	References
*Ocimum basilicum* (two varieties: Dark opal and Caesar)	anthocyanin phenols carotenoids	red:green:blue in equal proportion	[20]
*Ocimum basilicum* variety Caesar	phenols carotenoids	red:green:blue (4:1:1)	[20]
Red basilicum Green basilicum	antocyanin phenol	red:blue (2.3:1) suplemented with white red:blue (2.3:1)	[21]
Basil	phenol	R:B = 2:1 or 3:1	[23]
*Allium sativum*	phenol	red	[16]
*Coriandrum sativum*	phenol	blue	[26]
*Fagopyrum esculentum*	polyphenol flavonoid antioxidant activity	red blue	[74]
Hemp sprouts *Cannabis sativa*	antioxidant activity flavonoids polyphenols	green blue blue	[14]
*Melissa officinalis*	phenols	red and blue R:B = 2.3:1 + drought stress	[28]
*Triticum aestivum* sprouts	antioxidant capacity flavonoid	blue red	[13]
*Ocimum basilicum* L. (green- and purple-leaves) *Valerianella locusta* *Eruca sativa* L.	flavonoid	blue	[36]
*Vitis vinifera* (grape)	anthocyanins	blue	[37]
*Fagopyrum tataricum* (seedlings, sprouts)	anthocyanin	blue	[38]
*Cyclocarya paliurus* sprouts, wheel wingnut	anthocyanin	blue	[39]
*Agastache rugose*	rosmarinic acid tilianin (phenylpropanoids)	white	[40]
*Triticum aestivum* sprouts	epicatechin gallic acid and quercetin; decreased p-coumaric acid and epicatechin ferulic acid and p-coumaric acid	white blue red	[41]
*Anoectochilus roxburghii*	flavonoid	blue: red = 1:4	[42]
*Brassica napus* sprouts—Canola	glucosinolates phenolics	blue	[45]
Citrus juice sacs in vitro	carotenoid	blue	[46]
Chinese calapod callus	carotenoid	blue	[47]
Broccoli sprouts	glucosinolates	blue	[48]
*Myrtus communis* L. in vitro	phenolic compounds	red	[49]
Citrus	carotenoid	red	[50]
Buckwheat sprouts	carotenoid	white	[51]
Pea plants	carotene	red	[60]
Citrus red	cryptoxanthin	red	[61]
Tomatoes	lycopene	red	[62]
Broccoli sprouts	carotene violaxanthin	blue	[48]
Lemon basil (Ocimum × africanum)	carotenoid	red + blue	[63]
*Allium fistulosum* Welsh onions	carotenoid	white	[64]
*Thymus migricus*	thymol	blue	[65]
*Brassica oleraceae*	lutein	red	[66]
*Brassica oleracea* leaves	phenylacetaldehyde	red	[66]
*Petunia* hybrid	phenylpropanoid: 2-phenylethanol	red	[67]
*Mentha longifolia*	essential oil	red + blue	[68]
Rice leaves	antioxidant activity	blue + white + green	[69]
Red leaf lettuce	antioxidant activity	blue	[70]
*Anethum graveolens (dill)*	terpenoid	70% red + 10% blue	[71]
*Thymus migricus*	terpenoid	red and blue (R:B = 2.3:1)	[65]
Basil	terpenoid	red + blue R:B = 1:1.4,	[72]
*Mentha spicata*	terpenoid	red and green monochromatic	[73]

**Table 3:** The effect of LED on food preservation, postharvest losses.

Species	Effect	LED Light	References
Harvested red sweet pepper	maintained β-carotene, chlorophyll and lycopene for 7 days	red	[76]
Harvested yellow + green sweet peppers Harvested red sweet peppers	phenolic compounds, lignin precursors, flavanoids, coumarins phenolic compounds, lignin precursors, flavanoids, coumarins	red blue [76]	[76]
Avocado fruit	epicatechin	red	[77]
Tomatoes	lycopene, b-carotene, total phenol, flavonoid storage after harvest	red	[78]
Tomatoes	lycopene levels, chlorogenic acid, caffeic acid and rutin content after 7 days of storage	blue	[78]
*Coriandrum sativum* leaves during postharvest storage	total phenolic content mass loss reduction in color change increased antioxidant activities quercetin 2-tridecenal, 2-dodecenal, Z-9-19 hexadecenal (volatile compounds) reduced the loss of total chlorophyll constant b-carotene	blue blue and red blue (2 h) blue and red red	[79]
Outer and inner leaves lower Brussels sprouts postharvest conservation	respiration keeping the green color + visual quality, antioxidants in outer leaves, antioxidant capacity in outer leaves total flavonoids higher in outer than inner leaves on day 10 of storage	white-blue (WB)	[80]
Broccoli	delay in senescence total soluble sugars	white-blue (WB)	[81]
Lettuce	delay in senescence total soluble sugars	white-blue (WB)	[83,84]
Citrus	limited postharvest decay caused by *Penicillium*	single-spectrum blue	[85,86]
Roses	suppress sporulation of *Podosphaera pannosa*	blue	[89]
Grapevine leaves	inhibited lesion extension induced by *Botrytis cinerea* synthesis of stilbene	red	[90]
*Cucumis sativus*	salicilic acid synthesis resistance to *Sphaerotheca fuliginea*	red	[93]

## Data Availability

Not applicable.
